# Novel Urinary Glycan Biomarkers Predict Cardiovascular Events in Patients With Type 2 Diabetes: A Multicenter Prospective Study With 5-Year Follow Up (U-CARE Study 2)

**DOI:** 10.3389/fcvm.2021.668059

**Published:** 2021-05-24

**Authors:** Koki Mise, Mariko Imamura, Satoshi Yamaguchi, Mayu Watanabe, Chigusa Higuchi, Akihiro Katayama, Satoshi Miyamoto, Haruhito A. Uchida, Atsuko Nakatsuka, Jun Eguchi, Kazuyuki Hida, Tatsuaki Nakato, Atsuhito Tone, Sanae Teshigawara, Takashi Matsuoka, Shinji Kamei, Kazutoshi Murakami, Ikki Shimizu, Katsuhiro Miyashita, Shinichiro Ando, Tomokazu Nunoue, Michihiro Yoshida, Masao Yamada, Kenichi Shikata, Jun Wada

**Affiliations:** ^1^Department of Nephrology, Rheumatology, Endocrinology and Metabolism, Okayama University Graduate School of Medicine, Dentistry and Pharmaceutical Sciences, Okayama, Japan; ^2^Diabetes Center, Okayama University Hospital, Okayama, Japan; ^3^Center for Innovative Clinical Medicine, Okayama University Hospital, Okayama, Japan; ^4^Department of Chronic Kidney Disease and Cardiovascular Disease, Okayama University Graduate School of Medicine, Dentistry and Pharmaceutical Sciences, Okayama, Japan; ^5^Department of Diabetology and Metabolism, National Hospital Organization Okayama Medical Center, Okayama, Japan; ^6^Okayama Saiseikai General Hospital, Okayama, Japan; ^7^Kurashiki Central Hospital, Kurashiki, Japan; ^8^The Sakakibara Heart Institute of Okayama, Okayama, Japan; ^9^Japanese Red Cross Okayama Hospital, Okayama, Japan; ^10^Okayama City General Medical Center, Okayama, Japan; ^11^Nunoue Clinic, Tsuyama, Japan; ^12^GlycoTechnica Ltd., Yokohama, Japan

**Keywords:** cardiovascular event, diabetes, lectins, N-glycans, urinary biomarkers

## Abstract

**Background:** Although various biomarkers predict cardiovascular event (CVE) in patients with diabetes, the relationship of urinary glycan profile with CVE in patients with diabetes remains unclear.

**Methods:** Among 680 patients with type 2 diabetes, we examined the baseline urinary glycan signals binding to 45 lectins with different specificities. Primary outcome was defined as CVE including cardiovascular disease, stroke, and peripheral arterial disease.

**Results:** During approximately a 5-year follow-up period, 62 patients reached the endpoint. Cox proportional hazards analysis revealed that urinary glycan signals binding to two lectins were significantly associated with the outcome after adjustment for known indicators of CVE and for false discovery rate, as well as increased model fitness. Hazard ratios for these lectins (+1 SD for the glycan index) were UDA (recognizing glycan: mixture of Man5 to Man9): 1.78 (95% CI: 1.24–2.55, *P* = 0.002) and Calsepa [High-Man (Man2–6)]: 1.56 (1.19–2.04, *P* = 0.001). Common glycan binding to these lectins was high-mannose type of *N*-glycans. Moreover, adding glycan index for UDA to a model including known confounders improved the outcome prediction [Difference of Harrel's C-index: 0.028 (95% CI: 0.001–0.055, *P* = 0.044), net reclassification improvement at 5-year risk increased by 0.368 (0.045–0.692, *P* = 0.026), and the Akaike information criterion and Bayesian information criterion decreased from 725.7 to 716.5, and 761.8 to 757.2, respectively].

**Conclusion:** The urinary excretion of high-mannose glycan may be a valuable biomarker for improving prediction of CVE in patients with type 2 diabetes, and provides the rationale to explore the mechanism underlying abnormal *N*-glycosylation occurring in patients with diabetes at higher risk of CVE.

**Trial Registration:** This study was registered with the University Hospital Medical Information Network on June 26, 2012 (Clinical trial number: UMIN000011525, URL: https://upload.umin.ac.jp/cgi-open-bin/ctr_e/ctr_view.cgi?recptno=R000013482).

## Introduction

Cardiovascular disease (CVD) is a global burden especially in low- and middle-income countries and the leading cause of disability and mortality (1). The understanding of CVD risk factors is quite important to establish the cardiovascular risk prediction models. The age, gender, body mass index (BMI), systolic blood pressure (SBP), diabetes mellitus, smoking, total cholesterol levels, and past cardiovascular events are established and also traditional risk factors in middle-aged and older individuals (2). Chronic kidney disease (CKD) is an emerging global health burden with prevalence of ~15% of adult populations and is independently associated with increased cardiovascular event (CVE) including stroke and peripheral arterial disease (PAD) besides the traditional risk factors (3, 4). The addition of albuminuria and estimated glomerular filtration rate (eGFR) to traditional risk factors is significantly associated with cardiovascular outcomes in meta-analysis of general population cohort (5, 6). In type 2 diabetes, the CVE risk prediction is potentially improved by novel biomarkers involved in the biological process, not explained by the traditional risk factors (7). The improvement of risk prediction is statistically evaluated by discrimination ability and reclassification. The area under the receiver operating characteristic (AUROC) or c-index is a measurement for discrimination capacity of classification model, while the net reclassification improvement (NRI) is a commonly used measure for the prediction increment by the addition of new biomarkers. In the Second Manifestations of ARTertial disease (SMART) and the European Prospective Investigation into Cancer and Nutrition-NL (EPIC-NL) (8), Action in Diabetes and Vascular Disease: Preterax and Diamicron Modified Release Controlled Evaluation (ADVANCE) study (9), and the Outcome Reduction With Initial Glargine Intervention (ORIGIN) trial (10), the 23, 16, and 284 serum or plasma biomarkers were evaluated as to whether these biomarkers independently improve the AUROC and NRI, respectively. The three biomarkers in SMART/EPIC-NL, six in ADVANCE, and 10 in ORIGIN were identified in the prediction of CVD composite outcomes. N-terminal pro-B-type natriuretic peptide (NT-proBNP) was only the common biomarker in two studies for the prediction of composite CVE. In addition to the candidate approach for the identification of biomarkers, non-biased screening using metabolomic approach was also attempted such as amino acid (11) and lipid profiles (12).

The vigorous attempts were made for the identification of circulating biomarkers, and some of the urinary biomarkers were independently associated with CVE in patients with type 2 diabetes; however, they have failed to achieve significant incremental ability based on c-statistic and NRI (13–15). Urine albumin creatinine ratio (UACR) and eGFR are now regarded as the classical risk factors for CVE in type 2 diabetes; the concept of “cardiorenal syndrome” suggests that the identification of urinary biomarkers is promising approach. In the Urinary biomarker for Continuous And Rapid progression of diabetic nEphropathy (U-CARE) study, we performed urinary lectin microarray, measured urinary glycan signals binding to 45 lectins, and evaluated the potential for the prediction of 30% decline of eGFR or end-stage renal disease (ESRD) in the patients with type 2 diabetes (16). We found that the urinary glycan binding signals to *Sambucus nigra* (SNA), *Ricinus communis* (RCA120), *Dolichos biflorus* (DBA), *Agaricus bisporus* (ABA), *Artocarpus integrifolia* (Jacalin), and *Amaranthus caudatus* (ACA) improved the prediction of renal outcome in the models employing the known risk factors (16). The U-CARE study suggested that the global alterations of glycosylation of urinary protein are valuable disease progression markers and may be linked to disease mechanisms in diabetic kidney disease (DKD). The aim of this study (U-CARE Study 2) is to investigate in patients with type 2 diabetes the impact of urinary lectin microarray on the prediction of CVE by adding the glycan binding signals in the multivariate model containing the established risk factors of CVE.

## Materials and Methods

### Study Design and Participants

This is a second report of the U-CARE Study, a prospective cohort study, which started in 2012. Precise study design was described previously (16). In the current study, among 688 patients with type 2 diabetes admitted to multi-institutions in Japan, 680 patients were enrolled. Eight patients were excluded in this study since they were diagnosed with slowly progressive type 1 diabetes during follow-up. The diagnosis of diabetes was based on the Japanese Diabetes Society criteria (17). This study was registered with the University Hospital Medical Information Network in June 2012 (UMIN000011525). Written informed consent was obtained from all participants.

### Laboratory Parameters and Definitions

Urinary glycans were measured by the evanescent-field fluorescence-assisted lectin microarray (18). In brief, we measured urinary levels of Cy3-labeled glycoprotein binding to 45 lectins coated on microplates. In a previous study, we demonstrated that net glycan intensity [Net-I; raw glycan intensity (Raw-I)—background intensity] more accurately predicted the 24-h urinary glycan in comparison with Net-I or Raw-I/urinary creatinine ratios (16, 19). Based on the evidence, we analyzed glycan indexes defined by Net-I and logarithmically transformed Net-I when they did not follow normal distribution.

In this study, CVD was defined as events requiring admission for treatment, excluding the events with arrhythmia, dilated cardiomyopathy, and valvular heart disease to focus attention on the atherosclerotic cardiovascular diseases. Stroke was defined as cerebral bleeding and infarction requiring admission for treatment, while PAD as an event requiring admission for open surgery and/or endovascular intervention. CVE was defined as any CVD, stroke, or PAD events. Mortality due to cardiovascular death or other causes was also assessed. BMI was calculated as weight divided by the square of height (kg/m^2^). Hypertension was defined as a baseline blood pressure ≥140/90 mmHg or use of antihypertensive drugs. GFR was estimated by the Japanese coefficient-modified Chronic Kidney Disease Epidemiology Collaboration equation. The baseline UACR (mg/gCr) was measured in a spot urine specimen, and normoalbuminuria, microalbuminuria, and macroalbuminuria were defined as UACR < 30 mg/gCr, 30 ≤ UACR < 300 mg/gCr, and 300 mg/gCr ≤ UACR, respectively. Hemoglobin A1c (HbA1c) data are presented as National Glycohemoglobin Standardization Program values according to the recommendations of the Japanese Diabetes Society and the International Federation of Clinical Chemistry (20). The grade of diabetic retinopathy was determined by an ophthalmologist at baseline. The average annual values of clinical parameters including HbA1c, SBP, and diastolic blood pressure (DBP) were obtained. The administration of statin, angiotensin-converting enzyme (ACE) inhibitor or angiotensin II type I receptor blocker (ARB), glucagon-like peptide-1 receptor agonists (GLP1), and sodium glucose transporter 2 (SGLT2) inhibitor during follow-up were also recorded. These data and previous CVE were compared between patients with and without outcome.

### Study Endpoint

The primary endpoint was defined as incidence of CVE, and follow-up period was defined as the period from the initiation of observation to the earliest CVE, death, or last observation of clinical variables.

### Statistical Analysis

Data were presented as percentages or the mean ± standard deviation (SD), as appropriate. All skewed variables were subjected to natural logarithmic transformation to improve normality before analysis. Correlations among glycan indexes were evaluated by Pearson correlation analysis. The cumulative incidence rate of the primary outcome was estimated by Kaplan–Meier curves for urinary glycan quartiles in all patients, and incidence rates were compared with the log-rank test, including trend test among quartile groups. The Cox proportional hazards model was used to calculate the hazard ratio (HR) and 95% confidence interval (CI) for the event-censored endpoint. HR and 95% CI for the 1 SD increase of glycan index were individually calculated in each model. In the multivariate model, HRs were adjusted for age, gender, BMI, SBP, low-density lipoprotein (LDL) cholesterol, HbA1c, eGFR, and previous CVE at baseline. These covariates were selected as potential confounders on the basis of biological plausibility and previous reports (15, 21). False discovery rates (FDRs) for 45 glycan indexes were calculated by the Benjamini–Hochberg procedure in these Cox regression analyses to control the expected proportion of false rejections (22). The level of FDR was defined as 0.05. Time-dependent area under curve (AUC) in multivariate Cox regression analysis was obtained by integration of AUC in every 0.2 year from 0.5 year-observation calculated by 500 bootstrap sampling (23). We also compared Harrell's concordance index (c-index) between multivariate Cox proportional hazards models with or without glycan biomarkers. In addition, the Akaike information criterion (AIC) and Bayesian information criterion (BIC) in the multivariate Cox regression models were calculated to compare the model fitness. Furthermore, improvement in discriminating the 5-year risk of the study outcome was assessed by analyses of AUROC, category-free NRI, and absolute integrated discrimination improvement (IDI), as reported elsewhere (24, 25). The 95% CIs for the differences of the Harrell's c-index and AUROC, category-free NRI, and IDI were computed from 5,000 bootstrap samples to adjust for optimism bias. Two-tailed *P*-values < 0.05 were considered as statistically significant. Analyses and creation of graphs were performed with Stata SE software (version 14.0, StataCorp LP) and Origin (version 2018, OriginLab).

## Results

### Observation Period and Outcome Incidence

The median follow-up period was 4.8 years [interquartile range (IQR): 3.6–5.1 years]. During follow-up, the primary endpoint (CVE) occurred in 62 patients (9%), and 21 patients (3%) died. CVE was the cause of two patient deaths. Detailed information of CVE and other causes of death are shown in Supplementary Tables 1, 2.

### Clinical Characteristics

The clinical characteristics of all participants at baseline are displayed in Table 1. Their age was 63 ± 11 years (mean ± SD), 61% of the patients were men, and 24% of them had previous CVE. The median duration of diabetes was 11.1 years (IQR: 6.2–17.7), and baseline HbA1c was 7.1 ± 1.1% (54.3 ± 12.0 mmol/mol). Under 56% of statin use, the baseline LDL and non-high-density lipoprotein (non-HDL) cholesterol levels were 100.1 ± 25.3 and 126.5 ± 30.6 mg/dl, respectively. Similarly, 62% of the patients received antihypertensive agents, average blood pressures were SBP (131.0 ± 17.0 mmHg) and DBP (74.7 ± 10.9 mmHg). The mean baseline eGFR was 71.0 ± 17.7 ml/min/1.73 m^2^ and median UACR was 17.7 mg/gCr (IQR: 7.8–74.1). The average annual HbA1c, SBP, and DBP levels, and percentage of the use of ACE inhibitor or ARB, and GLP-1 receptor agonist during follow-up were not significantly different between the patients with and without outcome. Statin use during observation was significantly higher, and the use of SGLT2 inhibitor was significantly lower in patients with outcome compared with those without outcome (Supplementary Table 3).

**Table 1:** Baseline clinical parameters.

**Clinical parameters**		**All patients (*n* = 680)**
Age (years)		63 ± 11
Male (%)		61
BMI (kg/m^2^)		25.6 ± 4.6
Prior CVD/stroke/PAD (%)		17/9/1
Prior cardiovascular event (%)		24
Duration of DM (years)^*^		11.1 (6.2 > 17.7)
HbA1c	(%)	7.1 ± 1.1
	(mmol/mol)	54.3 ±12.0
Triglyceride (mg/dl)^*^		116 (81–163)
Total cholesterol (mg/dl)		180.5 ± 31.9
LDL cholesterol (mg/dl)		100.1 ± 25.3
Non-HDL cholesterol (mg/dl)		126.5 ± 30.6
Uric acid (mg/dl)		5.4 ± 1.4
SBP (mmHg)		131.0 ± 17.0
DBP (mmHg)		74.7 ± 10.9
Hypertension (%)^†^		70
Retinopathy (NDR/SDR/prePDR/PDR, %)^‡^		67/17/6/10
eGFR (ml/min/1.73 m^2^)		71.0 ± 17.7
CKD GFR Categories (G1/G2/G3a/G3b/G4/G5, %)		10/69/11/6/3/1
UACR (mg/gCr)^*^		17.7 (7.8–74.1)
Normo/Micro/Macro (%)		63/25/12
Any type of antihypertensive agents (%)		62
ACE inihibitor or ARB (%)		53
Calcium channel blocker (%)		38
Number of antihypertensive agents^*^		1 (0–2)
Treatment for diabetes		
(Diet only/OHA/Insulin, %)		4/64/32
Drug treatment for hyperglycemia		32/10/35/28/15/49/7
(SU/GLIN/BG/αGI/TZD/DPP4-I/GLP1, %)		
Drug treatment for dyslipidemia/statin use (%)		64/56

*BMI, body mass index; CVD, cardiovascular disease requiring admission for treatment; Stroke, cerebral bleeding or infarction requiring admission for treatment; PAD, peripheral arterial disease requiring admission for intervention or surgery; Cardiovasular event, any event of CVD, Stroke, and PAD; HbA1c, hemoglobin A1c; Duration of DM, estimated duration of diabetes mellitus; LDL cholesterol, low-density lipoprotein cholesterol; non-HDL cholesterol, non high-density lipoprotein cholesterol; SBP, systolic blood pressure; DBP, diastolic blood pressure; Retinopathy, diabetic retinopathy; NDR/SDR/prePDR/PDR, non diabetic retinopathy, simple diabetic retinopathy, pre proliferative diabetic retinopathy, and proliferative diabetic retinopathy, respectively; eGFR, estimated glomerular filtration rate, CKD GFR Categories; G1: ≥90 ml/min/1.73 m^2^, G2: 60–90 ml/min/1.73 m^2^, G3a: 45–59 ml/min/1.73 m^2^, G3b: 30–44 ml/min/1.73 m^2^, G4: 15–29 ml/min/1.73 m^2^; UACR, urinary albumin creatinine ratio; Normo/Micro/Macro, normoalbuminuria, microalbuminuria, and macroalbuminuria, respectively; ACE inhibitor or ARB, treatment with an angiotensin-converting enzyme inhibitor or angiotensin II type I receptor blocker, respectively; Diet only, diet regimen only; OHA, oral hypoglycemic agent; Insulin therapy, treatment with insulin (including basal-supported oral therapy); SU, sulfonylurea; GLIN, meglitinide anologs; BG, biguanide (Metformin); αGI, alpha-glucosidase inhibitors; TZD, thiazolidinediones; DPP4-I, DPP-4 inhibitors; GLP1, glucagon-like peptide 1 receptor agonists; SGLT2, sodium glucose transporter 2.*

* *Median (interquartile range)*.

† *Hypertension was defined as blood pressure ≥140/90 mmHg or any antihypertensive drug treatment*.

‡ *Data from 664 patients (98%) were available*.

### Relation Between Primary Endpoint and Glycan Binding to the Lectin Panel

**Figure 1:**
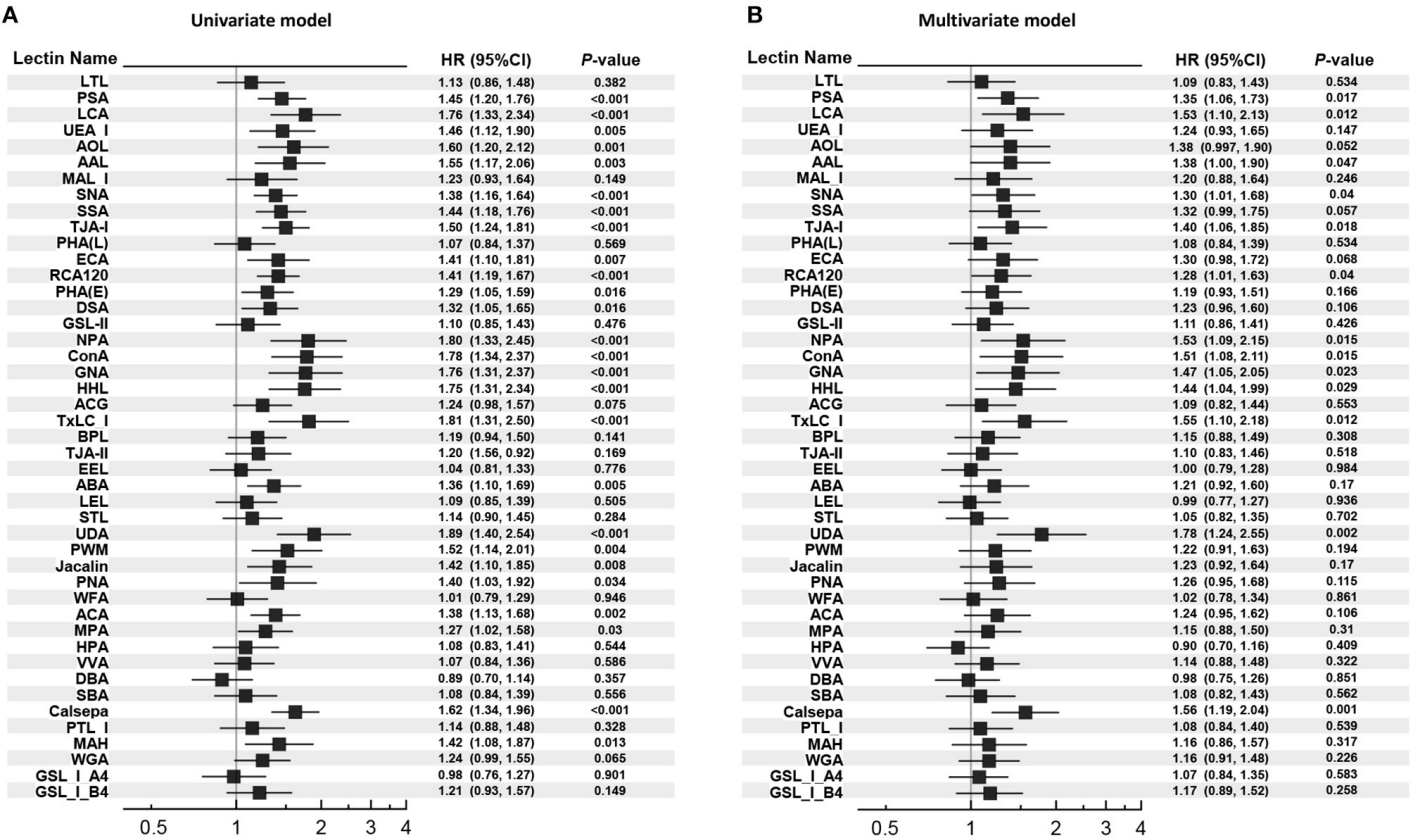
Univariate and multivariate Cox proportional hazard models for the outcome. **(A)** Univariate Cox proportional hazard models. **(B)** Multivariate Cox proportional hazard models. HR per 1 SD increase in each glycan index is shown. In the multivariate model, HR was adjusted for age, gender, body mass index, systolic blood pressure, hemoglobin A1c, low-density lipoprotein cholesterol, estimated glomerular filtration rate, past cardiovascular event at baseline. HR, hazard ratio; 95% CI, 95% confidence interval.

Unadjusted and adjusted HRs for glycan binding to the panel of 45 lectins with different specificities and the reported structure of the glycan binding to each lectin are shown in Figure 1 and Supplementary Table 4. The urinary glycan binding signals to 13 lectins [*Pisum sativum* (PSA), *Lens culinaris* (LCA), *Aleuria aurantia* (AAL), SNA, *Tanthes japonica* (TJAI), RCA120, *Narcissus pseudonarcissus* (NPA), *Canavalia ensiformis* (ConA), *Galanthus nivalis* (GNA), *Hippeastrum hybrid* (HHL), *Tulipa gesneriana* (TxLCI), *Urtica dioica* (UDA), and *Calystegia sepium* (Calsepa)] were significantly associated with the outcome in either of the univariate and multivariate models. Among them, both glycan binding signals to UDA and Calsepa were selected based on the FDR < 0.05 in the multivariate models. We fitted a series of multivariate Cox regression models, which include (i) only covariates, (ii) covariates + UACR, (iii) covariates + glycan signals (binding to UDA or Capsela), and (iv) covariates + UACR + glycan signal (Table 2). Then, the improvement of model fitness was evaluated based on the reduction of both AIC and BIC criteria. These criteria were minimized at model (iii) for both of UDA and Capsela, which were considered the best fitting model, that is, the two glycans were more substantially improved model fitness, and the addition of UACR did not exhibit improvement of model fitting. Glycan signals for UDA and Calsepa were not incorporated into the model at the same time to avoid multicolinearity because of the high correlation with each other (*r* = 0.87).

**Table 2:** Comparison of hazard ratio and model fitting between multivariate models with or without UACR and urinary glycans for UDA and Calsepa.

**Markers**	**Multivariate model**					**Markers**	**Multivariate model with UACR**				
	**HR**	**95% CI**	***P*-value**	**AIC**	**BIC**		**HR**	**95% CI**	***P*-value**	**AIC**	**BIC**
None	–	–	–	725.7	761.8	UACR	1.32	0.99–1.75	0.058	724.1	764.8
UDA	1.78	1.24–2.55	0.002	716.5	757.2	UDA	1.70	1.16–2.49	0.006	718.0	763.2
Calsepa	1.56	1.19–2.04	0.001	718.0	758.7	Calsepa	1.50	1.11–2.02	0.009	719.6	764.8

*Covariates in multivariate model: age, gender, body mass index, systolic blood pressure, hemoglobin A1c, low density cholesterol levels, estimated glomerular filtration rate, and past cardiovascular event at baseline. Each glycan index was employed into the multivariate model with or without log transformed UACR. UACR, urinary albumin creatinine ratio; HR, hazard ratio; 95% CI, 95% confidence interval; AIC, Akaike's information criterion; BIC, Bayesian information criterion; UDA, Urtica dioica; Calsepa, Calystegia sepium*.

The relationships between the glycan indexes and outcome remained largely unchanged when treated of statin, ACE inhibitor or ARB, and SGLT2 inhibitor during the follow-up period, and the average annual HbA1c, average annual SBP, and baseline non-HDL cholesterol were incorporated into the multivariate model (Supplementary Table 5). As shown in Supplementary Table 4, UDA and Calsepa are known to bind to a mixture of Man5 to Man9 and to High-Man (Man2-6), respectively. The common recognized glycans are classified into intermediate and immature products of *N*-glycan synthesis (26).

### Time-Dependent Area Under Curve and Harrell's C-Index in Cox Regression Model With or Without Urinary Glycans

**Figure 2:**
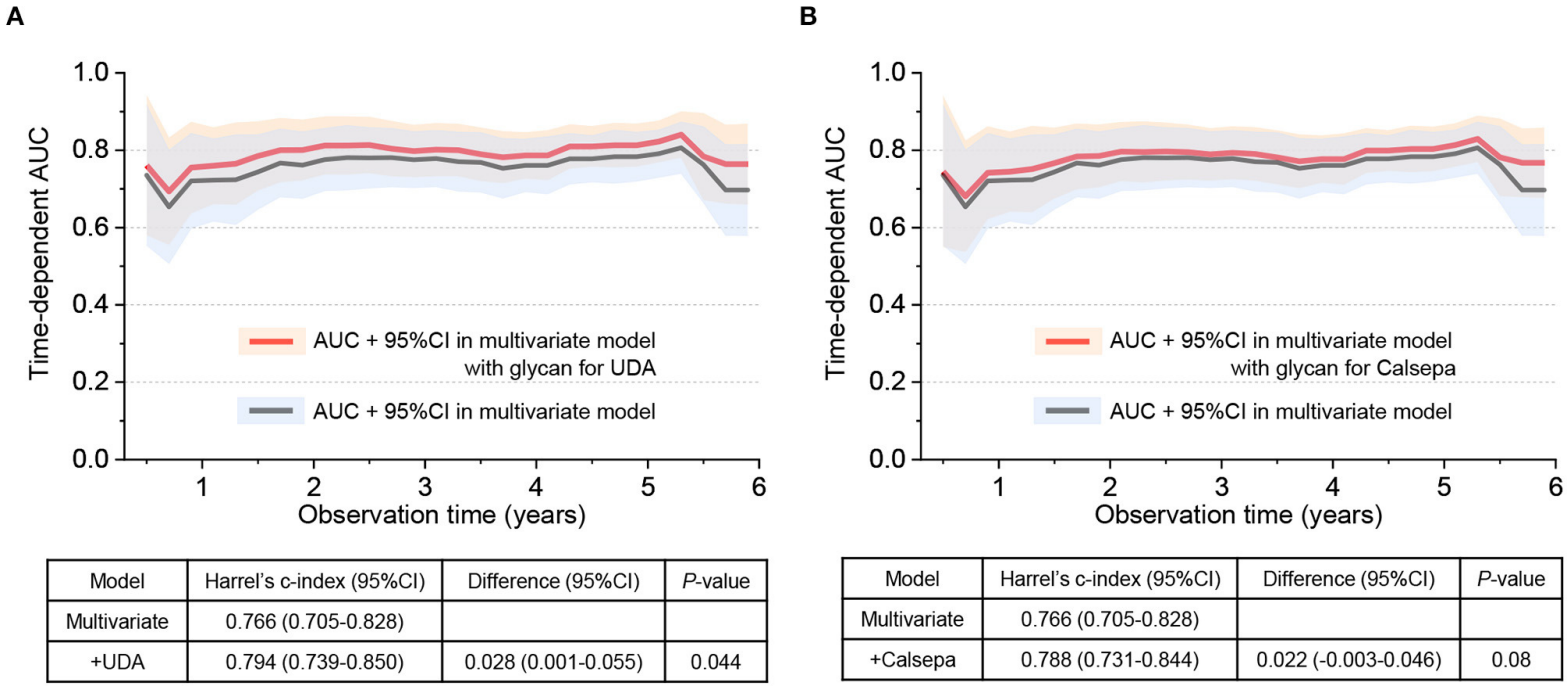
Time-dependent area under curve (AUC) and Harrell's C-index in Cox regression model with or without urinary glycans binding to UDA and Calsepa. **(A)** AUC and Harrell's C-index with or without urinary glycans binding to UDA. **(B)** AUC and Harrell's C-index with or without urinary glycans binding to Calsepa. In the multivariate Cox regression model without glycan, age, gender, body mass index, systolic blood pressure, hemoglobin A1c, low-density lipoprotein cholesterol, estimated glomerular filtration rate, past cardiovascular event at baseline were incorporated as adjusted variables. On the other hand, multivariate model with glycan includes the same covariates and any of two glycans binding to UDA and Calsepa. UDA, *Urtica dioica*; Calsepa, *Calystegia sepium*.

Time-dependent AUCs and Harrell's C indexes in multivariate Cox regression model with or without glycan binding signals to UDA and Calsepa are displayed in Figures 2A,B. Overall, AUCs during observation were higher in models with those glycan indexes than in model without them, while the Harrell's C-index was significantly higher only in the model containing glycan binding signal to UDA than in model without the glycans [Harrell's C-index for model without UDA: 0.766 (95% CI: 0.705–0.828), Harrell's C-index for model with UDA: 0.794 (0.739–0.850), and the difference in Harrell's C-index: 0.028 (0.001–0.055, *P* = 0.044)].

### Cumulative Incidence Rate of the Primary Outcome in Urinary Glycan Quartiles

**Figure 3:**
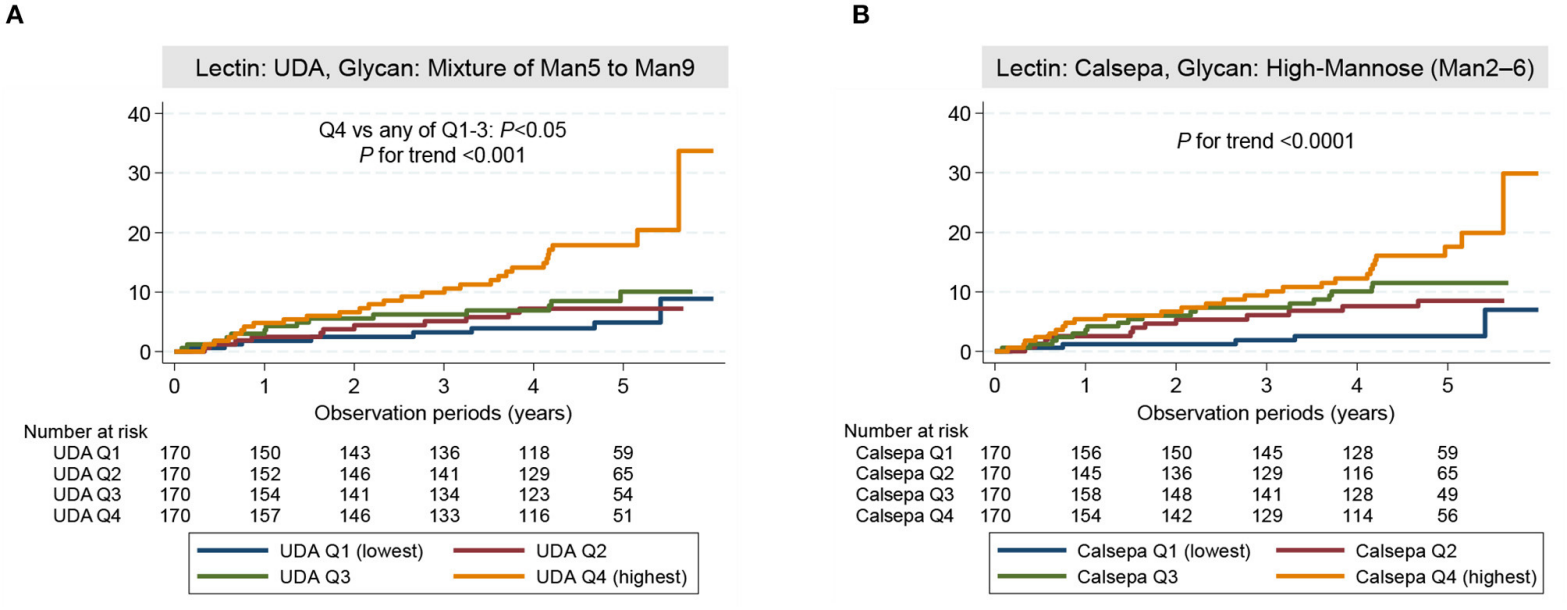
Cumulative incidence rate of the outcome. **(A)** Cumulative incidence rate in patients stratified according to the quartiles of urinary glycan indexes for UDA. **(B)** Cumulative incidence rate in patients stratified according to the quartiles of urinary glycan indexes for Calsepa. The cumulative incidence rate was significantly higher in patients with higher glycan indexes than in those with lower glycan indexes (UDA: *P* for trend < 0.001, Calsepa: *P* for trend < 0.0001). Among quartile groups for UDA, cumulative incidence rate was significantly higher in highest quartile group (Q4) compared with lower quartile groups (Q1–3) (*P* < 0.05). The log-rank test was used for failure analysis. UDA, *Urtica dioica*; Calsepa, *Calystegia sepium*; Man, Mannose.

Kaplan–Meier curves stratified according to quartiles for baseline urinary glycan binding to UDA and Calsepa are shown in Figure 3. The cumulative incidence rate of the outcome was significantly higher in the higher quartile for urinary glycan binding to UDA and Calsepa than in the lower quartiles [*P* for trend: < 0.001 for UDA (Figure 3A) and < 0.0001 for Calsepa (Figure 3B)].

### 5-Year Risk Classification Ability of Urinary Glycan Binding to Urtica Dioica and Calystegia Sepium

The difference of AUROC between logistic regression models with or without urinary markers, category-free NRI, absolute IDI for predicting the primary outcome at 5-year follow-up time obtained by adding UACR and the glycan indexes for UDA and Calsepa are summarized in Table 3. Adding of either glycan indexes to the multivariate model significantly improved the ability of discrimination and reclassification such as AUROC and NRI [difference in AUROC: 0.031 (95% CI: 0.001–0.062, *P* = 0.045) for UDA, 0.027 (0.001–0.053, *P* = 0.040) for Calsepa, category-free NRI: 0.368 (0.045–0.692, *P* = 0.026) for UDA, and 0.388 (0.099–0.677, *P* = 0.008) for Calsepa], whereas either of the two glycan indexes did not significantly improve integrated discrimination [IDI: 0.024 (−0.009–0.056, *P* = 0.16) for UDA and 0.021 (−0.010–0.053, *P* = 0.18) for Calsepa]. On the other hand, adding UACR did not show any significance on the incremental prediction [difference in AUROC: 0.017 (−0.002–0.035, *P* = 0.083), category-free NRI: 0.269 (−0.027–0.564, *P* = 0.075), and IDI: 0.005 (−0.014–0.024, *P* = 0.59)].

**Table 3:** AUROC, category-free NRI, and IDI for predicting the 5-year outcome with UACR and urinary glycan binding to UDA and Calsepa.

	**AUROC**	**Difference of AUROC**	***P*-value**	**Category-free NRI**	***P*-value**	**IDI**	***P*-value**
	**(95% CI)**	**(95% CI)**		**(95% CI)**		**(95% CI)**	
Only covariates	0.774						
	(0.711–0.837)						
With UACR	0.790	0.017	0.083	0.269	0.075	0.005	0.59
	(0.732–0.849)	(−0.002–0.035)		(−0.027–0.564)		(−0.014–0.024)	
With glycan to UDA	0.805	0.031	0.045	0.368	0.026	0.024	0.16
(Mixture of Man5 to Man9)	(0.748–0.862)	(0.001–0.062)		(0.045–0.692)		(−0.009–0.056)	
With glycan to Calsepa	0.801	0.027	0.040	0.388	0.008	0.021	0.18
[High-Man (Man2-6)]	(0.744–0.857)	(0.001–0.053)		(0.099–0.677)		(−0.010–0.053)	

*Covariates: age, gender, body mass index, systolic blood pressure, hemoglobin A1c, low density cholesterol levels, estimated glomerular filtration rate, and past cardiovascular event at baseline*.

*AUROC, The area under a receiver operating characteristic; NRI, net reclassification improvement; IDI, integrated discrimination improvement; UACR, urine albumin creatinine ratio; 95% CI, 95% confidence interval; UDA, Urtica dioica; Calsepa, Calystegia sepium; Man, Mannose*.

## Discussion

The urine glycan binding signals to UDA (mixture of Man5 to Man9) and Calsepa [High-Man (Man2-6)] improved model fitness scores for discrimination ability (Harrell's C index and AUROC), reclassification (NRI), and log-likelihood/complexity (AIC and BIC) when they were incorporated into the multivariate Cox and logistic regression model employing traditional risk factors. The strength of the current study was that the two urinary glycan signals were the novel urinary markers, which could provide the new mechanism of CVE in diabetes. They demonstrated the incremental predictive power with statistical significance, and they might be better markers than UACR. In previous studies of patients with type 2 diabetes, several urinary markers such as urinary kidney injury molecule 1, urinary neutrophil gelatinase-associated lipocalin, urinary liver-type fatty acid-binding protein, and urinary COOH-terminal propeptide of collagen VI, have been investigated for predicting CVE (13, 15, 27). However, none of them showed the statistical significance of model discrimination or reclassification in the multivariate model including known risk factors. Although it has been shown that UACR is associated with CVE independent of established confounders, its incremental predictive ability is limited (21). In our study, UACR had a marginal impact on the outcome in the multivariate Cox regression analysis [HR for logUACR: 1.32 (95% CI: 0.99–1.75), *P* = 0.058, Table 2], while it failed to demonstrate the significant values of AUROC, NRI, and IDI (Table 3) in the multivariate models, which was compatible with the previous results (21). In contrast to UACR, glycan indexes for UDA and Calsepa showed statistical significance of the incremental prediction as mentioned above. In addition, model fitness scores, i.e., AIC and BIC, were clearly better than that of UACR. Therefore, these novel glycan indexes might be superior to UACR for predicting CVE in patients with type 2 diabetes.

Interestingly, UDA and Calsepa recognize the high mannose glycan structures (Supplementary Figure 1). In endoplasmic reticulum (ER), Glc3Man9GlcNAc2 is transferred to the NXT/NXS sites of protein, Glc residues removed by glucosidases, and Man9GlcNAc2 converted to Man8GlcNAc2 by ER α-mannosidase I (MAN1B1). The glycoproteins are then transferred to *cis*-Golgi; the additional Man residues are removed until Man5GlcNAc2 is generated. Man5GlcNAc2 is a key intermediate for the pathway to hybrid and complex *N*-glycans in *trans*-Golgi and *trans*-Golgi network by the removal of mannose residues by Golgi mannosidases, while some of Man5GlcNAc2 also escapes further modification, and mature membrane or secreted glycoprotein carries Man5-9GlcNAc2, i.e., high mannose structures (Supplementary Figure 1A) (26). In the glycan analysis by urine lectin microarray, the elevation of high mannose and complex type of *N*-glycans in urine glycoproteins are tightly linked to the development of composite CVE.

The high-throughput plasma or serum *N*-glycan profiling studies using hydrophilic interaction liquid chromatography (HILIC) of peptide-*N*-glycosidase F digested and fluorescently labeled *N*-glycans were reported, and 46 *N*-glycan peaks (GP1-GP46) were demonstrated (28–32). In the patients with normo- and hyperglycemia during acute inflammation, the comparison of *N*-glycan profile demonstrated that increased branched, galactosylated, and sialylated tri- and tetraantennary *N*-glycans are associated with the development of type 2 diabetes (28). In Ghanaian population, branched, trigalactosylated, antennary fucosylated, and triantennary *N*-glycans (Supplementary Figure 1B) were increased in the patients with type 2 diabetes (29). A lower relative abundance of simple biantennary *N*-glycans and a higher abundance of branched, galactosylated, and sialylated complex *N*-glycans were increased both in type 1 (31) and type 2 (30) diabetes, and similar trends with increased levels of complex *N*-glycans (GP12, GP16, and GP22) were seen for higher UACR and greater annual loss of eGFR (29, 31). Recently, in the prospective European Prospective Investigation of Cancer (EPIC)—Potsdam cohort (*n* = 27,548), the increased levels of complex *N*-glycans, GP5 in women, and GP16, GP23, and GP29 in men, improved the accuracy of risk prediction score for CVD (32).

Independent of serum or plasma *N*-glycan profiling, our efforts to identify the biomarkers to improve the prediction of DKD and CVD outcomes have been directed to the clinical studies using urinary glycan profiling by lectin microarray in the patients with type 2 diabetes (16, 33). Previously, we found that urinary glycan profiling by lectin microarray demonstrated the considerable changes in glycan binding signals during the progression of DKD in urine samples rather than serum samples (16, 33). The changes in glycan profile in urine samples may reflect the glycosylation changes in glycoproteins produced in kidney tissues or the changes in selective permeabilities of blood-derived glycoproteins through glomerular capillaries. In addition, the lectins are long-standing experimental tools to identify the glycan structures, which enable lectin microarray to detect the broad range of glycans compared with HILIC or other methods using mass analysis. For instance, the capture of *O*-glycans and neutral *N*-glycans such as high-mannose type and hybrid type *N*-glycans (Supplementary Figure 1C) are extremely difficult in HILIC (34). Furthermore, only 20 μl of urine samples is required, and the single step of Cy3 labeling without enzymatic treatments achieve the less-time consuming and high-throughput analyses. By taking these advantages of urine lectin microarray, we successfully identified that the glycan binding signals to high mannose or mannose-recognizing lectins, UDA and Calsepa, contributed the improvement of the prediction models using established risk factors for CVE. In the previous study, we identified that the glycan-binding signals to SNA, RCA120, DBA, ABA, Jacalin, and ACA significantly improved the prediction models for 30% decline in eGFR or ESRD, and these lectins mainly recognized the *O*-glycan structures, suggesting the specificity of the analyses with lectin microarray (16). Furthermore, the application of those eight lectins for the urine samples of the patients with type 2 diabetes provides a useful diagnostic tool for the future risk of the CVD and DKD progression.

### Novel Mechanism of the Atherosclerotic Cardiovascular Event in Diabetes

The current clinical study provides the insight into the mechanism for the progression of atherosclerosis in type 2 diabetes. The detection of high mannose *N*-glycans, i.e., immature forms of *N*-glycans, in the urine samples in the patients with type 2 diabetes suggests the abnormalities in the processing and maturation of *N*-glycans in the ER and Golgi. In the ER, Glc1Man9GlcNAc2 *N*-glycans are properly folded by the assistance of calnexin and calreticulin, while the misfolded Man9GlcNAc2 is recognized by ER-degradation-enhancing α mannosidase I-like (EDEM) leading to ER degradation (26). The inhibition of ER α-mannosidase I (MAN1B1), which mediates the conversion of Man9GlcNAc2 to Man8GlcNAc2, was reported to enhance high mannose intercellular adhesion molecule-1 expression on endothelial cell surface (35). The impairment of quality control of glycoproteins and mannosidase activity in ER may cause the accumulation of high mannose *N*-glycans in ER. In addition, the knockout of the triple gene encoding Golgi α1,2-mannosidases (MAN1A1, MAN1A2, and MAN1B1) resulted in the production of high mannose *N*-glycans (36). The defects in the Golgi α1,2-mannosidases are also candidate mechanisms to produce high mannose *N*-glycans. The link between high mannose *N*-glycans and CVE further suggested the new mechanism for the progression of atherosclerosis in type 2 diabetes. High mannose *N*-glycans induced on endothelial cells by oscillatory shear stress, or tumor necrosis factor-α mediates the monocytic recruitment (37), and hypercholesterolemic patients exhibited higher plasma levels of a cluster of high-mannose and complex/hybrid *N*-glycans (38).

### Study Limitations

One of the key limitations in this study is that this was a multi-center observational study, and the therapeutic strategy of diabetes and its complications in each participant was not exactly standardized, which might have affected the incidence of the outcome. However, the sensitivity analyses revealed that the impact of glycan indexes for UDA and Calsepa on the outcome did not largely change even when the various treatment factors during follow-up periods were incorporated into the multivariate Cox regression models (Supplementary Table 5). In addition, we might not be able to adjust for other possible confounders in the multivariate models. Several blood biomarkers, such as NT-proBNP and high-sensitivity troponin T, have been established as useful markers for predicting CVE (9, 39). It remains unknown whether glycan indexes for UDA and Calsepa are significantly associated with the outcome independent of those biomarkers. Nevertheless, we hope that these novel urinary markers predict CVE independent of other confounders since these glycan markers could reflect the novel mechanism of CVE as mentioned above.

## Conclusions

The glycan profiling by urine lectin microarray demonstrated that the elevation of high mannose and complex type of *N*-glycans in urine glycoproteins is tightly linked to the development of CVE. UDA and Calsepa in lectin microarray may be a useful diagnostic tool for the prediction of CVD risk in patients with type 2 diabetes. The evidence linking the increased high mannose and complex type of *N*-glycans to the incidence of CVE in patients with diabetes suggests that the disease mechanisms and therapeutic targets are related to organellar dysfunction in the ER and Golgi, as well as to the progression of atherosclerosis.

## Data Availability Statement

The original contributions presented in the study are included in the article/Supplementary Material, further inquiries can be directed to the corresponding authors.

## Ethics Statement

The studies involving human participants were reviewed and approved by Institutional Review Boards of the Okayama University. The patients/participants provided their written informed consent to participate in this study.

## Author Contributions

KMis conceived the study, formulated the analysis plan, performed the statistical analyses, collected the clinical data, performed the urinary lectin microarray, and wrote the manuscript. MI collected and assessed all the clinical data. SY measured the urinary glycan binding signals to lectins and collected the clinical data. MW, CH, AK, SM, HU, AN, JE, KH, TN, AT, ST, TM, SK, KMu, IS, KMiy, SA, TN, and KS recruited the patients and assessed the data. MYo supported the statistical analyses. MYa measured the urinary glycan binding signals to lectins, analyzed the urinary lectin microarray data, and wrote the manuscript. JW conceived the study, supervised the data collection, analyzed the data, and edited the manuscript. All authors contributed to the interpretation of the data, critical revision of the manuscript, and approval of the final version of the manuscript.

## Conflict of Interest

MYa was a former employee of GP BioSciences Co., Ltd., and is currently an employee of GlycoTechnica Co., Ltd. JW received speaker honoraria from Astra Zeneca, Daiichi Sankyo, MSD, Novartis, Tanabe Mitsubishi, Taisho Toyama and received grant support from Baxter, Chugai, Dainippon Sumitomo, Ono, and Teijin. There are no other relevant declarations relating to employment, consultancy, patents, products in development, or marketed products. This does not alter the authors' adherence to all *Frontiers in Cardiovascular Medicine* policies on sharing data and materials. The remaining authors declare that the research was conducted in the absence of any commercial or financial relationships that could be construed as a potential conflict of interest.
