# Supplementary material for: Novel Urinary Glycan Biomarkers Predict Cardiovascular Events in Patients With Type 2 Diabetes: A Multicenter Prospective Study With 5-Year Follow Up (U-CARE Study 2)
Source: Front Cardiovasc Med. 2021 May 24;8:668059. doi: 10.3389/fcvm.2021.668059 (PMC8180567; doi:10.3389/fcvm.2021.668059)

Supplementary Table 1: Detailed information of cardiovascular events

Event		N
CVE		62
CVD		48
CVD	CABG	4
	CAD requiring PCI	34
	CAD requiring medication	7
	Congestive heart failure	3
Stroke		12
Stroke	Ischemic stroke	9
	Hemorrhagic stroke	3
PAD		4
PAD	Open surgery	2
	Endovascular intervention	2
3-Point MACE		22
4-Point MACE		28
4-Point Mace	Death from CVE	2
	Non-fatal myocardial infarction	9
	Non-fatal stroke	12
	Hospitalization for unstable angina	6

Two patients had both CVD and PAD events during follow-up.

Abbreviations: CVE, cardiovascular event; CVD, cardiovascular disease; CAD, coronary artery disease; PCI, Percutaneous coronary intervention; PAD, peripheral arterial disease; MACE, major adverse cardiac events.

Supplementary Table 2: Cause of death

**Cause of death**		**N**
Malignancy	Total case*	12
	Brain	1
	Lung	2
	Liver	2
	Pancreas	3
	Colon	1
	Prostate	1
	Bladder	1
	Leukemia/lymphoma	2
Acute myocardial infarction		1
Cerebral infarction		1
Bacterial pneumonia		1
Interstitial pneumonia		1
Alcoholic liver dysfunction		1
Cervical subluxation		1
Drowning		1
Unknown		2

*One patient had double primary cancer in lung and liver.

Supplementary Table 3: Comparisons of clinical parameters during follow-up and previous CVE between patients with and without outcome

Clinical parameters		Outcome (-) (n=618)	Outcome (+) (n=62)	*P*-value
Annual average HbA1c	(%)	7.1 ± 0.9	7.3 ± 1.2	0.15
	(mmol/mol)	54.5 ± 10.4	56.5 ± 13.1	
Annual average SBP		130.7 ± 13.3	132.4 ± 13.6	0.11
Annual average DBP		73.7 ± 8.5	72.3 ± 10.5	0.49
Statin use for more than half of follow-up period (%)		57	71	0.039
Treatment of ACE inhibitor or ARB (%)		54	63	0.17
Treatment of GLP-1 receptor agonists (%)		9	8	0.83
Treatment of SGLT2 inhibitor (%)		19	3	<0.01
Previous CVE (%)		20	60	<0.001

Treatment of statin, ACE inhibitor, ARB, and GLP-1 receptor agonist are defined as use of them for more than half of follow-up period, while SGLT2 inhibitor is defined as at least one time use during follow-up periods.
Abbreviations: CVE, cardiovascular event; HbA1c, hemoglobin A1c; SBP, systolic blood pressure; DBP, diastolic blood pressure; ACE, angiotensin-converting enzyme inhibitor; ARB, angiotensin II type I receptor blocker; GLP-1, glucagon-like peptide 1; SGLT2, sodium glucose transporter 2.

Supplementary Table 4: Preferred glycan structures binding to 45 lectins with different specificity

Lectin Name	Origin	Lectin Family	Monosaccharide Specificity	Preferred glycan structure (terminal epitope)
**LTL**	*Lotus tetragonolobus*	Legume (L-type)	Fuc	Fuc(α1-3)(Gal(β1-4))GlcNAc (Lex), Fuc(α1-2)Gal(β1-4)GlcNAc (H-type 2)
**PSA**	*Pisum sativum*	Legume (L-type)	Fuc/Man	Fuc(α1-6)GlcNAc, High-Man
**LCA**	*Lens culinaris*	Legume (L-type)	Fuc/Man	Fuc(α1-6)GlcNAc, High -Man
**UEA_I**	*Ulex europaeus*	Legume (L-type)	Fuc	Fuc(α1-2)Gal(β1-4)GlcNAc (H-type 2)
**AOL**	fungus, *Aspergillus oryzae*	Fucose lectin	Fuc	Fuc(α1-6)GlcNAc (core Fuc), Fuc(α1-2)Gal(β1-4)GlcNAc (H-type 2)
**AAL**	*Aleuria aurantia*	Fucose lectin	Fuc	Fuc(α1-6)GlcNAc (core Fuc), Fuc(α1-3)(Gal(β1-4))GlcNAc (Lex)
**MAL_I**	*Maackia amurensis*	Legume (L-type)	Sia	Sia(α2-3)Gal(β1-4)GlcNAc
**SNA**	*Sambucus nigra*	Ricin B-cahin-like (R-type)	Sia	Sia(α2-6)Gal/GalNAc
**SSA**	*Sambucus sieboldiana*	Ricin B-cahin-like (R-type)	Sia	Sia(α2-6)Gal/GalNAc
**TJA-I**	*Trichosanthes japonica*	Ricin B-cahin-like (R-type)	Sia	Sia(α2-6)Gal/GalNAc
**PHA(L)**	*Phaseolus vulgaris*	Legume (L-type)	Complex	Tri/tetra-antennary complex-type *N*-glycan
**ECA**	*Erythrina cristagalli*	Legume (L-type)	Gal	Gal(β1-4)GlcNAc
**RCA120**	*Ricinus communis*	Ricin B-cahin-like (R-type)	Gal	Gal(β1-4)GlcNAc
**PHA(E)**	*Phaseolus vulgaris*	Legume (L-type)	Gal	*N*-glycans with outer Gal and bisecting GlcNAc
**DSA**	*Datura stramonium*	Hevein (Chitin-type)	GlcNAc	(GlcNAc(β1-4))n, triantennary, tetraantennary *N*-glycans
**GSL-II**	*Griffonia simplicifolia*	Legume (L-type)	GlcNAc	Agalactosylated tri/tetra antennary glycans, GlcNAc
**NPA**	*Narcissus pseudonarcissus*	Monocot (GNA-related)	Man	High-Man including Man(α1-6)Man
**ConA**	*Canavalia ensiformis*	Legume (L-type)	Man	High-Man including Man(α1-6)(Man(α1-3))Man
**GNA**	*Galanthus nivalis*	Monocot (GNA-related)	Man	High-Man including Man(α1-3)Man
**HHL**	*Hippeastrum hybrid*	Monocot (GNA-related)	Man	High-Man including Man(α1-3)Man or Man(α1-6)Man
**ACG**	*Agrocybe cylindracea*	Galectin	Gal	Sia(α2-3)Gal(β1-4)GlcNAc
**TxLC_I**	*Tulipa gesneriana*	Monocot (GNA-related)	Man/GalNAc	Man(α1-3)(Man(α1-6))Man, bi- and tri-antennary *N*-glycans, GalNAc

**BPL**	*Bauhinia purpurea alba*	Legume (L-type)	Gal	Gal(β1-3)GalNAc, GalNAc
**TJA-II**	*Trichosanthes japonica*	Others	Gal	Fuc(α1-2)Galβ1, GalNAcβ1
**EEL**	*Euonymus europaeus*	Legume (L-type)	Gal	Gal(α1-3)Gal(β1-4)GlcNAc, Fuc(α1-2)(Gal(α1-3))Gal(β1-4)GlcNAc
**ABA**	fungus, *Agaricus bisporus*	Others	Gal, GlcNAc	Gal(β1-3)GalNAc, GlcNAc
**LEL**	tomato, *Lycopersicon esculentum*	Hevein (Chitin-type)	GlcNAc	(GlcNAc(β1-4))n, (Gal(β1-4)GlcNAc)n (polylactosamine)
**STL**	potato, *Solanum tuberosum*	Hevein (Chitin-type)	GlcNAc	(GlcNAc(β1-4))n, (GlcNAc(β1-4)MurNAc)n (peptidoglycan backbone)
**UDA**	*Urtica dioica*	Hevein (Chitin-type)	GlcNAc	GlcNAc(β1-4)GlcNAc, Man5 to Man9
**PWM**	pokeweed, *Phytolacca americana*	Hevein (Chitin-type)	GlcNAc	(GlcNAc(β1-4))n
**Jacalin**	*Artocarpus integrifolia*	Jacalin	Gal	Gal(β1-3)GalNAc, αGalNAc (6O-unsubstituted)
**PNA**	peanut, *Arachis hypogaea*	Legume (L-type)	Gal	Gal(β1-3)GalNAc
**WFA**	*Wisteria floribunda*	Legume (L-type)	GalNAc	GalNAc(β1-4)GlcNAc, Gal(β1-3(-6))GalNAc
**ACA**	*Amaranthus caudatus*	Ricin B-cahin-like (R-type)	Gal	Gal(β1-3)GalNAc
**MPA**	*Maclura pomifera*	Jacalin	Gal	Gal(β1-3)GalNAc, GalNAc
**HPA**	snail, *Helix pomatia agglutinin*	Discoidin	GalNAc	αGalNAc
**VVA**	*Vicia villosa*	Legume (L-type)	GalNAc	αGalNAc, GalNAc(α1-3)Gal
**DBA**	*Dolichos biflorus*	Legume (L-type)	GalNAc	Blood group A antigen, GalNAc(α1-3)GalNAc
**SBA**	soybean, *Glycine max*	Legume (L-type)	GalNAc	GalNAc, GalNAc(α1-3)Gal
**Calsepa**	*Calystegia sepium*	Jacalin	Man	High-Man (Man2–6), *N*-glycans including bisecting GlcNAc
**PTL_I**	*Psophocarpus tetragonolobus*	Legume (L-type)	GalNAc	αGalNAc
**MAH**	*Maackia amurensis*	Legume (L-type)	Sia	Sia(α2-3)Gal(β1-3)(Sia(α2-6))GalNAc
**WGA**	wheat germ, *Triticum unlgaris*	Hevein (Chitin-type)	GlcNAc	(GlcNAc(β1-4))n, NeuAc
**GSL_I_A4**	*Griffonia simplicifolia Lectin I Isolectin A4*	Legume (L-type)	GalNAc	αGalNAc
**GSL_I_B4**	*Griffonia simplicifolia Lectin I Isolectin B4*	Legume (L-type)	Gal	αGal

Abbreviations; Fuc, Fucose; Gal, Galactose; GlcNAc, N-acetylglucosamine ; Man, Mannose; Sia, Sialic acid; GalNAc, N-acetylgalactosamine.

Supplementary Table 5: Sensitivity analysis of multivariate Cox regression model

Glycan Marker	Model 1			Model 2			Model 3		
	HR	95% CI	*P*-value	HR	95% CI	*P*-value	HR	95% CI	*P*-value
UDA	1.78	1.24-2.55	0.002	1.80	1.25-2.59	0.001	1.84	1.27-2.65	0.001
Calsepa	1.55	1.19-2.04	0.001	1.57	1.20-2.07	0.001	1.56	1.19-2.05	0.001
**Glycan Marker**	**Model 4**			**Model 5**			**Model 6**		
	**HR**	**95% CI**	***P*-value**	**HR**	**95% CI**	***P*-value**	**HR**	**95% CI**	***P*-value**
UDA	1.76	1.23-2.51	0.002	1.83	1.27-2.64	0.001	1.78	1.24-2.55	0.002
Calsepa	1.55	1.18-2.04	0.001	1.60	1.21-2.10	0.001	1.55	1.19-2.04	0.001

Covariates of Model 1: variables in multivariate model (age, gender, body mass index, SBP, HbA1c, LDL cholesterol, estimated glomerular filtration rate, and past cardiovascular event at baseline) plus statin use for more than half periods of follow-up
Covariates of Model 2: variables in multivariate model plus ACE inhibitor or ARB use for more than half periods of follow-up
Covariates of Model 3: variables in multivariate model plus SGLT2 inhibitor use during follow-up
Covariates of Model 4: variables in multivariate model except for HbA1c plus annual average of HbA1c
Covariates of Model 5: variables in multivariate model except for SBP plus annual average of SBP
Covariates of Model 6: variables in multivariate model except for LDL cholesterol plus non-HDL cholesterol
HRs were shown as values for SD increase of glycan index.
Abbreviations: HR: hazard ratio, 95% CI: 95% confidence interval; UDA, *Urtica dioica*; Calsepa, *Calystegia sepium*; SBP, systolic blood pressure; HbA1c, hemoglobin A1c; LDL, low-density lipoprotein; ACE, angiotensin-converting enzyme inhibitor; ARB, angiotensin II type I receptor blocker; SGLT2: sodium glucose transporter 2; HDL, non-high density lipoprotein.

Supplementary Figure 1: UDA is known to bind to mixture of Man5 to Man9, and Calsepa to High-Man (Man2-6).

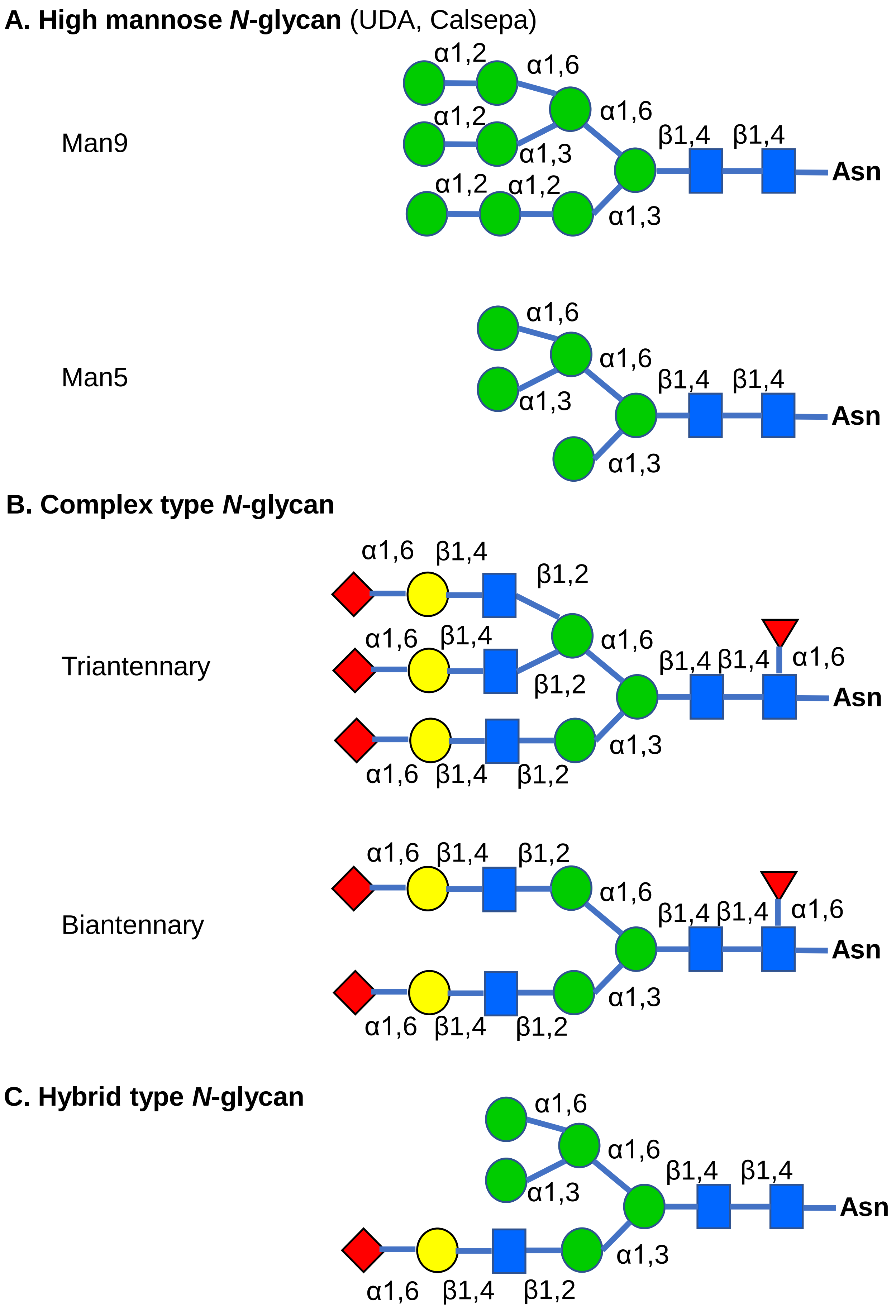

Supplement: Supplementary file 1 [file Data_Sheet_1.PDF]
